# Confidence in Dementia Management and Its Associated Factors among Primary Care Doctors in Malaysia

**DOI:** 10.3390/ijerph19169995

**Published:** 2022-08-13

**Authors:** Nurul Izzah Sodri, Mohamed-Syarif Mohamed-Yassin, Mariam Mohamad, Noorhida Baharudin

**Affiliations:** 1 Department of Primary Care Medicine, Faculty of Medicine, Universiti Teknologi MARA, Selayang Campus, Batu Caves 68100, Selangor, Malaysia; 2 Department of Public Health Medicine, Faculty of Medicine, Universiti Teknologi MARA, Sungai Buloh Campus, Jalan Hospital, Sungai Buloh 47000, Selangor, Malaysia

**Keywords:** dementia, confidence, primary care, Malaysia, general practitioner, knowledge, attitudes

## Abstract

Primary care doctors (PCDs) play an important role in the early diagnosis and management of dementia. This study aimed to determine the knowledge, attitude, and confidence in managing dementia among PCDs in Malaysia. It also aimed to determine the factors associated with higher confidence levels in dementia management. An online-based cross-sectional study using *Google Forms*^TM^ was performed. Sociodemographic and work-related data were collected, and Dementia Knowledge among General Practitioners & General Practitioners Attitude and Confidence Scale for Dementia questionnaires were utilized to assess the knowledge, attitude, and confidence scores. Multiple linear regression was conducted to determine the association between sociodemographic factors, knowledge, and attitude with the confidence in dementia management score. A total of 239 PCDs participated, with the majority being female (72.4%) and Malay (64.4%) and working in public primary care clinics (67.4%). The mean (±SD) score for confidence was 2.96 (±0.76). Among the factors associated with higher confidence levels in managing dementia were higher dementia knowledge scores, higher attitude towards dementia scores, prior dementia education, and the availability of nearby referral services for dementia. Malaysian PCDs’ confidence in dementia management was comparable to the PCDs of other countries. Strategies addressing these factors should be implemented to improve the confidence of PCDs in managing dementia.

## 1. Introduction

Dementia is currently the fifth leading cause of death globally, accounting for 2.4 million deaths in 2016 [1]. It is also one of the major causes of disability and dependency among the elderly worldwide [2]. The estimated total global societal cost of dementia was USD 1.3 trillion in 2019, with the main cost of care contributed by formal and informal social care [3]. In Malaysia, the 2018 National Health and Morbidity Survey found that the overall prevalence of probable dementia was 8.5% among older adults aged 60 and above [4,5]. Dementia is also among the top ten causes for disability-adjusted life years (DALYs) among Malaysians aged 75 and above [6]. 

Primary care doctors (PCDs) are often the first point of contact for many elderly patients with early symptoms or signs of dementia. Hence, they play an important role in the recognition of dementia. They need to have a high index of suspicion of this condition and should screen for it when encountering patients with subjective memory complaints. The timely diagnosis of dementia by PCDs will ideally lead to referral to an appropriate specialist (geriatric psychiatrist, psychiatrist, geriatrician, neurologist) or a multidisciplinary memory clinic. Then, appropriate medications can be started, and psychosocial interventions can be instituted [7]. 

However, many studies have reported that there is a low detection rate of dementia in primary care settings [8,9,10,11,12]. The dementia diagnosis rate in primary care setting in the United Kingdom (UK) was reported to be 40% in 2010 [13]. A meta-analysis on the global prevalence and determinants of undetected dementia in 2017 suggested increased risks of undetected dementia by PCDs [14]. A special report by the American Alzheimer’s Association in 2020 highlighted the increasing burden on PCDs to cope with the demands of care for people living with dementia due to the shortage of dementia care specialists, as well as the growing number of people living with dementia [15]. As the frontliners of this crisis, many PCDs recognize the need to provide dementia care effectively but feel underprepared and inadequately trained [15]. PCDs have been found to have barriers in managing dementia due to the gaps in their knowledge, skills, attitudes, and resources in dementia care [12,16,17,18,19]. A United Kingdom (UK) study highlighted that one-third of general practitioners expressed limited confidence in their diagnostic skills, whilst two-thirds lacked confidence in managing behavioral and other problems related to dementia [20]. In terms of barriers to the diagnosis and management of patients with dementia in primary care, a systematic review by Koch & Iliffe et al. highlighted that PCDs had concerns about their own abilities to make a diagnosis of or manage a patient with dementia. The lack of confidence and inherent stigma about making a wrong diagnosis and disclosing the diagnosis with empathy may lead to PCDs’ reluctance or inability to recognize dementia earlier [19,21]. This is supported by the theory of planned behavior, in which perceived behavioral control, or a person’s confidence in his or her own ability, might affect the person’s behavior or intention to adopt a certain behavior [22].

Therefore, it is important to study which factors might affect PCDs’ confidence in managing dementia. Several factors have been found to affect the level of confidence in dementia management among PCDs. Subramaniam et al. found that general practitioners (GP) in Singapore with postgraduate qualification were associated with a higher confidence in dealing with patients with dementia and their caregivers [23]. A study among GPs in Beijing reported a positive association between the attitudes and self-confidence in dementia care skills [24].

In Malaysia, primary care doctors consist of those working in public, private, and university primary care clinics. The majority of PCDs in Malaysia do not possess a postgraduate qualification in family medicine [25]. These groups of PCDs are called medical officers if they work in a public clinic and general practitioners if they work in a private clinic. PCDs with postgraduate qualifications in Family Medicine are referred to as Family Medicine Specialists, and most of them work in public health clinics and university primary care clinics [25]. PCDs in Malaysia provide general medical care to patients and are responsible for overall care, including the coordination of medical specialists and supportive care [25,26].

To date, there is no published study on the levels of knowledge, attitude, and confidence in dementia management among PCDs in Malaysia. Previous studies related to this topic were conducted among pharmacists and university students [27,28]. Hence, this study had several objectives. First, this study aimed to determine the knowledge, attitude, and confidence levels in managing dementia among PCDs in Malaysia. Next, it aimed to compare the knowledge, attitude, and confidence levels in managing dementia between PCDs with and without postgraduate qualification. Finally, this study aimed to test the hypothesis that there is an association between sociodemographic and work-related factors, as well as dementia knowledge and attitudes, and PCDs’ confidence in managing dementia.

## 2. Materials and Methods

### 2.1. Study Design and Population

This was a cross-sectional study among PCDs in Malaysia using an online questionnaire. Those with only a basic medical degree were defined as PCDs without a postgraduate qualification in Family Medicine (PCD-noPG-Qual); those with a postgraduate qualification in Family Medicine, such as a Diploma in Family Medicine, Master of Family Medicine, Fellow of the Royal Australian College of General Practitioners (FRACGP), or Member of the Royal College of General Practitioners (MRCGP) UK, were defined as PCDs with postgraduate qualifications (PCD-PG-Qual). The study population was PCDs in Malaysia who had been practicing in a primary care setting for at least one year, either in a public, private, or university clinic. These PCDs were approached through the three largest PCD associations in Malaysia, which were the Malaysian Family Medicine Specialists’ Association (FMSA), the Academy of Family Physicians Malaysia (AFPM), and the Malaysian Primary Care Network (MPCN), and received an invitation via email and/or social media, which included a link to the questionnaire. Only those who fulfilled the inclusion and exclusion criteria and consented to the study were included. The inclusion criteria were PCDs who were registered with the Malaysian Medical Council and had one or more years of working experience in a Malaysian primary care setting. PCDs who were no longer actively practicing in a primary care setting in Malaysia for the past 6 months and those who were working in primary care clinics only as locums were excluded.

### 2.2. Study Tool

The questionnaire consisted of four main parts: sociodemographic and work-related information, assessment of knowledge on dementia, assessment of attitude towards dementia, and assessment of confidence in dementia management. 

The sociodemographic data collected included age, gender, ethnicity, and highest medical qualification, while the work-related information collected was duration and place of practice, the availability of a referral center nearby one’s place of practice, and previous exposure to dementia such as a history of providing professional services to people living with dementia. 

The Dementia Knowledge among General Practitioners (DKAGP) questionnaire by Pentzek et al. was used to assess the participants’ knowledge on dementia. It contains 20 multiple choice questions on dementia epidemiology, genetics, diagnosis, symptoms, therapy, management, and support [29]. It has a Cronbach alpha value of 0.73 for internal consistency. One point is awarded for each correct answer, and zero points are awarded for an incorrect answer. Thus, the minimum score is 0, and the maximum score is 20. Higher scores indicate better knowledge.

For the assessment of attitude and confidence in dementia management among PCDs, the General Practitioners Attitude and Confidence Scale for Dementia (GPACS-D), a validated questionnaire with a Cronbach α value of 0.77, was utilized [30]. This 15-item questionnaire is divided into three subscales, which are confidence in clinical abilities (α = 0.81), attitude to care (α = 0.77), and engagement (α = 0.45). The attitude and confidence subscales were used in the current study. There are six items each in the confidence and attitude subscales and three items in the engagement subscale. Each item is measured via a five-point Likert scale (1 = strongly disagree, 2 = disagree, 3 = neutral, 4 = agree, 5 = strongly agree). Each subscale has a minimum score of one and a maximum score of five, as the total score for each domain was divided by the number of items in the scale. Higher scores indicate a better attitude and higher confidence in dementia management [30]. The permission to use these questionnaires was obtained from their developers. The questionnaires were then converted into an online version (*Google Forms*^TM^), which was pre-tested among five PCDs to ensure that it was functioning well. These PCDs reported that the online questionnaire was easy to complete and that the items were easy to understand. These five PCDs were not invited for the actual study.

### 2.3. Sample Size Calculation

To achieve the objective of determining the association between sociodemographic and work-related factors and confidence in managing dementia, multiple linear regression was used in the analysis of this study. The sample size recommended for studies using multiple linear regression is 237, based on a simulation analysis by Kelley and Maxwell [31].

### 2.4. Participant Recruitment, Sampling Method, and Data Collection

The sampling method for this study was convenience sampling until the target sample size was achieved. This method was chosen in view of the study being conducted using an online questionnaire in the form of *Google Forms*^TM^ due to the movement restriction order imposed by the Malaysian government during the Coronavirus disease 2019 (COVID-19) pandemic. Participant recruitment was carried out between February 2021 and August 2021. The council members of the Malaysian Family Medicine Specialists’ Association, the Academy of Family Physicians Malaysia, and the Malaysian Primary Care Network were approached and requested to send all their members an invitation through email and social media messaging services, which included a link to the study information sheet. This was followed by a consent form, which must have been completed before proceeding to the online questionnaire. Reminders were sent after a 1-month and 2-month interval from the initial invitation.

### 2.5. Data Entry and Statistical Analysis

Data entry and statistical analysis were performed using the latest IBM^®^ Statistical Package for Social Sciences (SPSS) software (IBM Corp., Armonk, NY, USA) [32]. Descriptive statistics were used to describe the sociodemographic and work-related characteristics. Normality testing was conducted for continuous variables using the Kolmogorov–Smirnov test. Means [+/− Standard Deviations (SD)] were used to describe continuous data with normal distribution, while non-normally distributed data were presented as medians and interquartile ranges (IQR). Frequencies and percentages were used to describe the categorical data. 

The knowledge, attitude, and confidence in dementia management scores were also presented using descriptive statistics. An independent T-test was used to compare the mean knowledge, attitude, and confidence scores between PCDs with postgraduate qualifications and those without postgraduate qualifications. Statistical significance was taken at a *p* value < 0.05.

Inferential analysis was conducted to determine the association between sociodemographic and work-related factors, knowledge, and attitude and the confidence scores. Univariate analysis was performed using simple linear regression to identify factors with a *p* value of <0.05. These factors were then analyzed using multiple linear regression to control for any confounding factors. The backward method was used for the analysis. The model assumptions of the linearity of the numerical independent variables and the normality and equal variance of the residuals were checked. Statistical significance was taken at a *p* value < 0.05.

## 3. Results

### 3.1. Characteristics of Respondents

**Figure 1:**
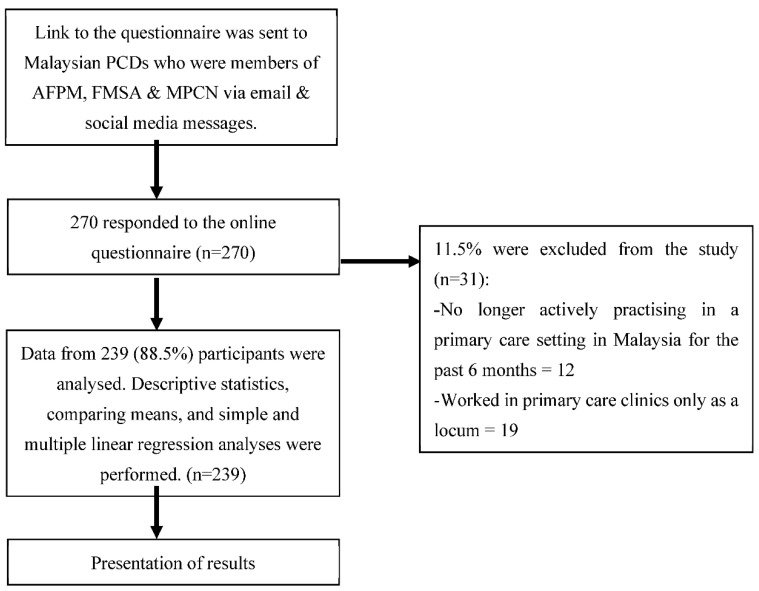
Flow chart of the study.

A total of 270 primary care doctors responded to the online questionnaire. However, only 239 (88.5%) fulfilled the inclusion and exclusion criteria and consented to the study (Figure 1). From the total of 239 PCDs, the median age was 34 years old (IQR 6). The majority of the respondents were female (72.4%) and Malay (64.4%) and worked in public primary care clinics (67.4%) (Table 1). Among the PCDs who participated, 137 (57.3%) did not have postgraduate qualifications in Family Medicine (PCD-noPG-Qual).

### 3.2. Knowledge, Attitude, and Confidence in Dementia Scores among Primary Care Doctors

Table 2 shows the mean knowledge, attitude, and confidence in dementia management scores of Malaysian PCDs with and without postgraduate qualifications. The mean knowledge score of all PCDs was 10.25 (SD ± 4.09). The mean knowledge score was significantly higher in PCDs with postgraduate qualifications (PCD-PG-Qual) compared to those without postgraduate qualifications (PCD-noPG-Qual) (12.34, SD ± 3.35 vs. 8.69, SD ± 3.90; t(232) = 7.77, *p* < 0.001).

The overall mean attitude to dementia score was 4.30 (SD ± 0.40), with a slightly higher score in PCD-PG-Qual (4.34, SD ± 0.42) compared to PCD-noPG-Qual (4.27, SD ± 0.40). However, this difference was not statistically significant (*p* = 0.143).

The overall mean confidence in dementia management score was 2.96 (SD ± 0.76). The PCD-PG-Qual group had a significantly higher mean confidence in dementia management score compared to the PCD-noPG-Qual group (3.28, SD ± 0.63 vs. 2.73, SD ± 0.77; t(235) = 6.08, *p* < 0.001).

### 3.3. Factors Associated with Confidence in Dementia Management

Table 3 shows the results of the univariate analysis using simple linear regression. From this analysis, all the factors had a significant *p* value of <0.05, except for working in private clinics. All of these significant factors were included in the multiple linear regression analyses.

Table 4 shows the factors associated with confidence in dementia management among PCDs. Based on the multiple linear regression analyses, seven factors were significantly associated with the confidence in dementia management score, explaining 45% of the variation (R^2^ = 0.45). The seven factors were gender, place of practice, availability of referral service for dementia nearby one’s place of practice, provided professional services for people living with dementia, prior dementia education, knowledge on dementia score, and attitude towards dementia care score. All assumptions (linearity, independence, normality of the response variable, homoscedascity, and fit of independent numerical variable) for the multiple linear regression were met. Female PCDs had lower confidence scores compared to males (b = −0.25 (95% CI: −0.42, −0.09). Otherwise, the following factors were associated with higher confidence scores: practiced in a university primary care clinic (b = 0.31, 95% CI: 0.08, 0.54), had referral service for dementia nearby place of practice (b = 0.23, 95% CI: 0.04, 0.41), provided professional services for people living with dementia (b = 0.42, 95% CI: 0.26, 0.58), and prior dementia education (b = 0.18, 95% CI: 0.02, 0.34). An increase in the knowledge score by 1 (b = 0.06, 95% CI: 0.04, 0.08) would increase the confidence score by 0.06. Similarly, an increase in the attitude score by 1 (b = 0.35, 95% CI: 0.16, 0.53) would increase the confidence score by 0.35.

## 4. Discussion

This study found that the mean knowledge on dementia score of PCDs in Malaysia was 10.25 (SD ± 4.09) out of a maximum possible score of 20. The mean confidence score was significantly higher in PCDs with postgraduate qualifications (3.28, SD ± 0.63) compared to those without such qualifications (2.73, SD ± 0.77; t (235) = 6.08, *p* < 0.001). This finding is similar to that in a study by Subramaniam et al. in 2018, which found that general practitioners in Singapore with both a medical degree and a Master of Medicine (Family Medicine) degree were associated with a higher confidence in managing people living with dementia and their caregivers compared to those with only a medical degree [23]. Another study done by Mason et al. in 2019 found that GP supervisors in Australia were significantly more confident in their in clinical abilities to manage dementia than GP registrars [30].

Interestingly, from the multiple linear regression analyses performed, postgraduate qualification was found to be not significantly associated with a higher confidence in managing dementia. Instead, there were several other significant factors that were associated with a higher confidence in managing dementia. One of these include PCDs who were practicing in university primary care clinics. They were found to be more confident in dementia management compared to those practicing in public health clinics. This might be because many university clinics are staffed by academic Family Medicine Specialists and Family Medicine postgraduate trainees who have more opportunities for dementia education and exposure to people living with dementia. 

Other important factors that resulted in a higher confidence in dementia management included receiving prior dementia education in the past year (in the form of seminars, continuing medical education courses, etc.), previously providing professional services for people living with dementia, and having referral services for dementia nearby their place of practice. This finding is similar to that in a study in Australia by Mason et al., which found that PCDs who had provided professional services to people with dementia scored higher in confidence compared to those who had never treated clients with dementia [30]. Another study by Mason et al. on the effects of a dementia education intervention on the attitudes and confidence of GPs in Australia also reported a significant increase in the confidence scores of GPs between the pre-test and post-test periods [33]. A cohort study done in 2013 among 29 PCDs in North Carolina, United States reported a significantly higher overall confidence in their dementia care competency six months after a one-day training session on dementia screening, diagnosis, and management that included direct engagement with local support service providers [34]. The study also found improvements in these PCDs’ ability to educate patients and caregivers about dementia and in making appropriate referrals to community care services after the training [34]. 

In the current study, female PCDs had a lower confidence in managing dementia score compared to males. Similarly, gender differences in confidence had been reported among GPs in the UK, where female GPs were less likely to say that they were confident in diagnosing dementia and in giving advice for managing dementia-related symptoms [35]. The reasons for this are not clear but could possibly be linked to their previous experience as medical students; the literature reported that female medical students are less confident in their own abilities [36]. Furthermore, given the self-reporting nature of this study, gender differences in self-report response biases should be considered [37,38,39,40]; female PCDs might be more willing to admit that they lack confidence in managing dementia.

This study also found that an increase in the knowledge score would increase the confidence score. Similarly, an increase in the attitude score would increase the confidence score. This is consistent with the study by Wang et al., who reported a positive association between the scores of attitude and self-confidence in dementia care skills [24]. Another study in Germany also found a positive association between attitude towards dementia care and confidence or self-reported competence [41]. In concordance with our study, a recent study conducted among Malaysian PCDs also found that knowledge was positively associated with confidence in managing in-flight emergencies [42]. These findings further support the importance of continuing professional development (CPD) activities for doctors. In line with this, the Malaysian Medical Regulations 2017, which replaced the Medical Regulations 1974, made the requirement of 20 CPD points compulsory for Malaysian medical practitioners to renew their annual practicing certificate [43].

### 4.1. Strengths and Limitations of This Study

The strength of this study is that it is the first study to determine the knowledge, attitude, and confidence in dementia management and the factors associated with higher confidence in dementia management scores among PCDs in Malaysia. However, there are some limitations to this study. First, due the cross-sectional study design, the findings could only show an association but not a causal relationship between knowledge, attitude, and the various sociodemographic factors and confidence in dementia management score. Next, convenience sampling via an online recruitment method was chosen for the reasons of practicability and feasibility, given the uncertainties regarding movement restrictions due to the COVID-19 pandemic. Thus, this nonprobability sampling method may be prone to sampling bias. Therefore, efforts were made to minimize this bias by ensuring the invitations reached as many PCDs as possible via the three largest PCD associations in the country, various online and social media platforms, and repeated reminders. Thirdly, there were other factors that may influence the confidence in dementia management which were beyond the scope of this study, such as patient outcomes and caregiver feedback. Thus, the results from the multiple linear regression should be interpreted based on the variables included in this regression model only.

### 4.2. Implications for Clinical Practice and Future Research

The findings from this study provided insight into the factors affecting the confidence in managing dementia among PCDs in Malaysia. A more holistic approach to dementia education intervention via CPD activities in the form of training and courses can be conducted to improve the knowledge and attitudes towards dementia care, thus increasing the confidence in dementia management [44]. This training may also include updated information on the dementia care services available, with clear dementia management pathways to guide PCDs involved in coordinating care for people living with dementia [45,46]. 

The recent publication of the updated Clinical Practice Guidelines on Management of Dementia in Malaysia will also help to improve dementia education among PCDs [7]. Policymakers can also contribute by providing funding for education and training to increase the specialty workforce through scholarships and sponsored programs [15]. In fact, a postgraduate diploma in Primary Care for the Elderly was recently launched in a local private university and was partially sponsored by the university to encourage participation among PCDs [47]. Improved coordinated care with dementia care specialists in nearby referral centers is also needed for PCDs to cater to the increasing demand of people living with dementia [46,48]. A recent study that discussed potential challenges for the delivery of dementia care in Malaysia highlighted a lack of coordination, collaboration, and communication between healthcare provisions and suggested better integration among multi-professional groups, including allied health professionals and non-governmental organizations, to enhance dementia care [46]. 

The findings from this study should serve as a baseline for future research in exploring other factors that may be associated with confidence in dementia management. Furthermore, a pre- and post-study can be conducted among PCDs to evaluate the effectiveness of a dementia education program. 

## 5. Conclusions

In conclusion, the knowledge, attitude, and confidence in dementia management among Malaysian primary care doctors were comparable to PCDs of other countries. Several sociodemographic factors, which included prior dementia education, the availability of nearby referral services for dementia, and improved knowledge and attitudes towards dementia care scores, were found to be positively associated with confidence in managing dementia. Strategies addressing these factors should be implemented to improve the confidence of PCDs in managing dementia and, subsequently, the outcomes for people living with dementia.

## Figures and Tables

**Table 1:** Sociodemographic details of study participants.

Characteristics of the Participants	Postgraduate Qualification in Family Medicine *		Total, *n* = 239 **
	With, *n* = 102	Without, *n* = 137	
**Age (years)**			
Median (IQR)	36.5 (7)	32 (5)	34 (6)
**Gender, *n* (%)**			
Male	31 (47.0)	35 (53.0)	66 (27.6)
Female	71 (41.0)	102 (59.0)	173 (72.4)
**Ethnicity, *n* (%)**			
Malay	52 (33.8)	102 (66.2)	154 (64.4)
Chinese	28 (58.3)	20 (41.7)	48 (20.1)
Indian	18 (60.0)	12 (40.0)	30 (12.6)
Other	4 (57.1)	3 (42.9)	7 (2.9)
**Duration of practice, *n* (%)**			
5 years and below	13 (14.1)	79 (85.9)	92 (38.5)
6–10 years	58 (58.6)	41 (41.4)	99 (41.4)
>10 years	31 (64.6)	17 (35.4)	48 (20.1)
**Place of practice, *n* (%)**			
Public health clinic	78 (48.4)	83 (51.6)	161 (67.4)
Private clinic	15 (30.6)	34 (69.4)	49 (20.5)
University primary care clinic	9 (31.0)	20 (69.0)	29 (12.1)
**Availability of referral service for dementia near place of practice, *n* (%)**			
Yes	93 (52.2)	85 (47.8)	178 (74.5)
No	6 (20.7)	23 (79.3)	29 (12.1)
Not sure	3 (9.4)	29 (90.6)	32 (13.4)
**Provided professional services for people living with dementia, *n* (%)**			
Yes	73 (62.4)	44 (37.6)	117 (49.0)
No	29 (23.8)	93 (76.2)	122 (51.0)
**Have family member with dementia, *n* (%)**			
Yes	31 (48.4)	33 (51.6)	64 (26.8)
No	71 (40.6)	104 (59.4)	175 (73.2)
**Received dementia education within the past 1 year, *n* (%)**			
Yes	49 (49.0)	51 (51.0)	100 (41.8)
No	53 (38.1)	86 (61.9)	139 (58.2)

* Data presented as row percentage. ** Data presented as column percentage. IQR—interquartile range.

**Table 2:** Mean knowledge, attitude, and confidence in dementia management scores of PCDs and comparison between PCD-PG-Qual and PCD-noPG-Qual.

Domain	Mean (±SD) Score of Total PCD	Mean (±SD) Score of PCD-PG-Qual *	Mean (±SD) Score of PCD-noPG-Qual **	Mean Difference of Scores (95% CI)	t Value (df)	*p* Value
**Knowledge on dementia score (0–20)**	10.25 (±4.09)	12.34 (±3.35)	8.69 (±3.90)	3.65 (2.72–4.58)	7.77 (232)	**<0.001**
**Attitude to clinical abilities score (1–5)**	4.30 (±0.40)	4.34 (± 0.42)	4.27 (±0.40)	0.08 (0.03–0.18)	1.47 (237)	0.143
**Confidence in dementia score (1–5)**	2.96 (±0.76)	3.28 (±0.63)	2.73 (±0.77)	0.55 (0.37–0.73)	6.08 (235)	**<0.001**

* PCD-PG-Qual—Primary care doctors with postgraduate qualifications. ** PCD-noPG-Qual—Primary care doctors without postgraduate qualifications. **Emboldened**: Statistical significance at *p* < 0.05.

**Table 3:** Simple linear regression analyses on the factors associated with confidence in dementia management among PCDs.

Characteristics	b (95% CI)	Standard Error (SE)	*p* Value
**Age**	0.02 (0.01, 0.03)	0.01	**0.005**
**Gender**			
• Male	Ref		
• Female	−0.23 (−0.45, −0.01)	0.11	**0.037**
**Ethnicity**			
• Non-Malay	Ref		
• Malay	−0.30 (−0.50, −0.10)	0.10	**0.003**
**Postgraduate qualification in Family Medicine**			
• Without postgraduate qualification	Ref		
• With postgraduate qualification	0.55 (0.37, 0.74)	0.09	**<0.001**
**Duration of practice**	0.02 (0.01, 0.03)	0.01	**0.009**
**Place of practice**			
• Public health clinic	Ref		
• Private clinic	−0.14 (−0.38, 0.11)	0.12	0.272
• University primary care clinic	0.43 (0.14, 0.73)	0.15	**0.004**
**Availability of referral service for dementia nearby place of practice**			
• No	Ref		
• Yes	0.62 (0.41, 0.83)	0.11	**<0.001**
**Provided professional services for people living with dementia**			
• No	Ref		
• Yes	0.68 (0.50, 0.85)	0.09	**<0.001**
**Have family member with dementia**			
• No	Ref		
• Yes	0.22 (0.00, 0.44)	0.11	**0.049**
**Prior dementia education**			
• No	Ref		
• Yes	0.48 (0.29, 0.66)	0.10	**<0.001**
**Knowledge on dementia score**	0.10 (0.07, 0.12)	0.01	**<0.001**
**Attitude towards dementia care score**	0.49 (0.26, 0.72)	0.12	**<0.001**

Ref—reference group; **Emboldened**: Statistical significance at *p* < 0.05.

**Table 4:** Multiple linear regression analyses on the factors associated with confidence in dementia management among PCDs.

Characteristics	Adjusted b (95% CI)	t Statistics	Standard Error (SE)	*p* Value
**Gender**				
• Male	Ref			
• Female	−0.25 (−0.42, −0.09)	−3.02	0.08	**0.003**
**Place of practice**				
• Public health clinic	Ref			
• Private clinic	−0.03 (−0.25, 0.18)	−0.31	0.11	0.756
• University primary care clinic	0.31 (0.08, 0.54)	2.64	0.09	**0.009**
**Availability of referral service for dementia nearby place of practice**				
• No	Ref			
• Yes	0.23 (0.04, 0.41)	2.37	0.08	**0.018**
**Provided professional services for people living with dementia**				
• No	Ref			
• Yes	0.42 (0.26, 0.58)	5.19	0.08	**<0.001**
**Prior dementia education**				
• No	Ref			
• Yes	0.18 (0.02, 0.34)	2.19	0.08	**0.029**
**Knowledge score**	0.06 (0.04, 0.08)	5.33	0.01	**<0.001**
**Attitude score**	0.35 (0.16, 0.53)	3.72	0.09	**<0.001**

R^2^ = 0.45. MLR backward method. Model assumptions of the linearity of the numerical independent variables and the normality and equal variance of the residuals were met. No multicollinearity or interaction problems between independent variables. Emboldened: Statistical significance at *p* < 0.05. Ref—reference group.

## Data Availability

The datasets generated and analyzed during the current study are available from the corresponding author on reasonable request and are subjected to data protection laws and regulations.
